# Development of Microsatellite Markers for *Tanacetum cinerariifolium* (Trevis.) Sch. Bip., a Plant with a Large and Highly Repetitive Genome

**DOI:** 10.3390/plants11131778

**Published:** 2022-07-05

**Authors:** Filip Varga, Zlatko Liber, Jernej Jakše, Ante Turudić, Zlatko Šatović, Ivan Radosavljević, Nina Jeran, Martina Grdiša

**Affiliations:** 1 Department of Seed Science and Technology, Faculty of Agriculture, University of Zagreb, Svetošimunska c. 25, 10000 Zagreb, Croatia; fvarga@agr.hr (F.V.); zsatovic@agr.hr (Z.Š.); njeran@agr.hr (N.J.); mgrdisa@agr.hr (M.G.); 2 Centre of Excellence for Biodiversity and Molecular Plant Breeding (CoE CroP-BioDiv), Svetošimunska c. 25, 10000 Zagreb, Croatia; aturudic@agr.hr (A.T.); ivan.radosavljevic@biol.pmf.hr (I.R.); 3 Department of Biology, Faculty of Science, University of Zagreb, Marulićev trg 9a, 10000 Zagreb, Croatia; 4 Department of Agronomy, Biotechnical Faculty, University of Ljubljana, Jamnikarjeva 101, 1000 Ljubljana, Slovenia; jernej.jakse@bf.uni-lj.si

**Keywords:** *Tanacetum cinerariifolium*, Asteraceae, genomic SSRs

## Abstract

Dalmatian pyrethrum (*Tanacetum cinerariifolium* (Trevis.) Sch. Bip.) is an outcrossing plant species (2n = 18) endemic to the eastern Adriatic coast and source of the natural insecticide pyrethrin. Due to the high repeatability and large genome (1C-value = 9.58 pg) our previous attempts to develop microsatellite markers using the traditional method were unsuccessful. Now we have used Illumina paired-end whole genome sequencing and developed a specific procedure to obtain useful microsatellite markers. A total of 796,130,142 high-quality reads (approx. 12.5× coverage) were assembled into 6,909,675 contigs using two approaches (de novo assembly and joining of overlapped pair-end reads). A total of 31,380 contigs contained one or more microsatellite sequences, of which di-(59.7%) and trinucleotide (25.9%) repeats were the most abundant. Contigs containing microsatellites were filtered according to various criteria to achieve better yield of functional markers. After two rounds of testing, 17 microsatellite markers were developed and characterized in one natural population. Twelve loci were selected for preliminary genetic diversity analysis of three natural populations. Neighbor-joining tree, based on the proportion of shared alleles distances, grouped individuals into clusters according to population affiliation. The availability of codominant SSR markers will allow analysis of genetic diversity and structure of natural Dalmatian pyrethrum populations as well as identification of breeding lines and cultivars.

## 1. Introduction

The Balkans is a well-known European hotspot of plant biodiversity with a large number of aromatic and medicinal plant species distributed across the peninsula [1]. One such species is Dalmatian pyrethrum (*Tanacetum cinerariifolium* (Trevis.) Sch. Bip.; Asteraceae, Anthemideae) that inhabits dry grasslands with shallow, rocky soils [2] along the eastern Adriatic coast and is easily recognized during the flowering season by its flower heads, which consist of white petal-like ray florets at the edges and yellow disk florets in the centre of the flower head [3]. Pyrethrum plants produce pyrethrins (pyrethrin I and II, cinerin I and II, jasmolin I and II), which have a *knock-down* effect due to disruption of the insect’s nervous system, followed by paralysis and death [4]. Pyrethrins are one of the most commercially used plant insecticides and the species has a long history of cultivation and domestic and agricultural use, with records of commercial cultivation in Dalmatia dating back to 1850s [5]. This region remained the world’s leading producer of pyrethrum until World War I. By then, production was introduced in other countries such as Japan [6] and Kenya [7], from where it spread further. Production in Croatia (part of Yugoslavia at the time) gradually declined and eventually ceased altogether, in part due to the discovery and widespread use of DDT [8]. Today, the largest producers of pyrethrum are concentrated in East Africa (Tanzania, Rwanda, and Kenya), Oceania (Papua New Guinea) [9] and Australia (Tasmania) [10].

The pyrethrin content and composition of Croatian natural Dalmatian pyrethrum populations have been largely investigated, especially in the last 15 years. Pyrethrin content reaches up to 1.35% of flower dry weight [11,12,13] which is lower than in commercial lines such as pyrethrum grown in Australia, where pyrethrin content can reach up to 2.5% of flower dry weight [14].

While pyrethrin content has been well studied in natural pyrethrum populations, far fewer studies have been conducted on the genetic diversity of *T. cinerariifolium* [15,16]. In addition, to date, no studies have explored the association between genetic and biochemical diversity in this species. One of the first studies attempted to clarify the relationship between ploidy level and certain morphological traits of Dalmatian pyrethrum in order to easily distinguish diploids from triploids based on morphology [17]. Recent research has focused more on identifying candidate genes involved in the biosynthesis of pyrethrin compounds, such as the chrysanthemyl diphosphate synthase gene [18,19,20,21] and genes involved in the in the 2-C-methyl-D-erythritol 4-phosphate pathway (MEP) pathway [22]. Another study that focused on the variation of tandem repeats in subtelomeres between individuals of this species showed high polymorphism of subtelomeres based on detailed FISH (fluorescence in situ hybridization) analysis [16]. A draft genome of the species was also constructed, revealing some genes potentially encoding enzymes specific for pyrethrin biosynthesis [23]. To date, only one study on the genetic diversity of natural populations of Dalmatian pyrethrum has been conducted [15]. In this comprehensive study (20 populations sampled along the Croatian Adriatic coast) based on Amplified Fragment Length Polymorphism (AFLP) markers, researchers discovered high gene diversity and high number of private alleles in the northern Adriatic populations, which gradually decreased towards the south, most likely due to historical overexploitation.

Microsatellite markers (single sequence repeats—SSRs) are more informative than AFLPs because they are codominant, locus-specific and more reproducible [24]. They are often used as molecular markers in population genetics due to the high level of polymorphism [25,26]. The presence of highly repetitive sequences and the large genome size have caused the development of SSR markers for this species to fail, as has been the case for species with similar genome features when a traditional approach consisting of restriction digestion, hybridization, library construction, cloning and Sanger sequencing has been used [27,28,29]. The advent of next generation sequencing (NGS) has enabled the efficient generation of a large amount of genome sequence data from which microsatellite primers can be identified and has significantly reduced the cost and time required for microsatellite marker development [30]. Compared to other plant species native to the Balkans that are agriculturally exploited (oregano, Dalmatian sage), genetic research on Dalmatian pyrethrum is severely underdeveloped. At the time of writing, only 418 nucleotide records for this species are available in NCBI (24 January 2022). Genomic characterization of this species using codominant molecular markers is urgently needed not only from the perspective of plant genetic resources conservation, but also for utilization in breeding programs aimed at reviving commercial production of Dalmatian pyrethrum in Croatia.

The objective of this research was to develop SSR markers by means of Next Generation Sequencing, which can then be used in the population genetics study of *T. cinerariifolium*.

## 2. Results and Discussion

### 2.1. Contigs Assembly and Genome-Wide Identification of SSR Markers

A total of 796,130,142 high-quality reads (120 Gbp in total) were obtained by next generation sequencing of *T. cinerariifolium* with a GC content of 35.89%. Low GC content was previously observed in species with rather small or very large genomes such as *Elaeagnus angustifolia* L. (35.0%), *Trifolium striatum* L. (35.6%) [31] and *Helichrysum italicum* (Roth) G. Don (34.1%) [32]). The fact that GC base synthesis requires more biochemical cost than AT synthesis may be one of the main reasons why larger genomes tend to have lower GC content [33]. On the other hand, the prevalence of AT-rich motifs and low frequency of GC-rich motifs is the characteristic of dicotyledonous species compared to monocotyledonous species [34].

The first approach (de novo assembly of subset of the data) resulted in 923,207 contigs. The total length of the assembled contigs was 409.5 Mbp. The length of the longest contig was 82,765 bp and the N50 value of the assembly was 451 bp. The assembly had a mean GC content of 34.34%. The second approach (assembly of overlapping pair-end reads) yielded 5,986,468 sequences with a total length of 1311 Mbp (representing 13.7% of the estimated genome size) and a mean GC content of 34.41% (Table 1).

**Figure 1:**
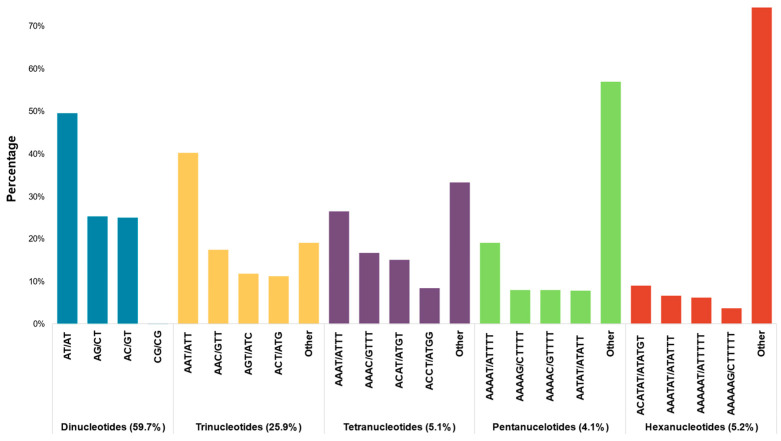
Frequency of SSR repeat motifs in *T. cinerariifolium* assembly.

Screening of the obtained contigs (6,909,675) for microsatellites without mononucleotide repeats revealed 35,556 microsatellite loci in 31,380 SSR-containing contigs. The majority of SSR-containing contigs (28,108—89.6%) contained only one microsatellite locus, while 3272 (10.4%) contained more than one microsatellite locus (Table 1). Dinucleotide repeats were the most common (nearly 60% of the remaining loci), followed by trinucleotide repeats (more than a quarter of the remaining loci). Tetra-, penta-, and hexanucleotides had similarly low frequencies (Figure 1). These results are consistent with other species from the Asteraceae family such as *Conyza canadensis* (L.) Cronquist. [35], *Cynara cardunculus* var. *scolymus* L. [36] and *H. italicum* [32]. The most common dinucleotide motifs were AT/AT, accounting for 29.6% of the total repeats, followed by AG/CT (15.1%) and AC/GT (15%). Among trinucleotides, AAT/ATT (10.4%) was the most common motif, followed by AAC/GTT (4.5%) and AGT/ATC (3.1%) (Figure 1).

### 2.2. Selection, Testing, and Characterization of SSR Markers

The 35,556 identified microsatellite loci were selected using described filtering criteria. Only di- and trinucleotide motifs were considered, as well as loci with GC content similar to average assembly GC content (43 ± 10%). Sequences with majority reads mapped with multi-mapping occurrences or sequences with average coverage not close to sequencing coverage (12.5×) were also excluded, as were sequences with hits to repetitive elements occurrences, as identified by RepeatMasker. Filtering of the microsatellite loci according to the selection criteria resulted in 1006 sequences that were checked for the number of occurrences against the draft genome of *T. cinerariifolium* [23], which further narrowed the selection to 56 sequences that were used for primer design. These 56 primer pairs were tested on five samples from different populations. Even 39 of them were excluded from further analysis because they were not polymorphic between samples, more than two alleles were detected in the same sample, or PCR products were completely absent. The remaining 17 (30.4%) microsatellites that showed both good amplification and polymorphism were selected for further testing on 20 samples from one Dalmatian pyrethrum population (MAP02806). Similar approach using various multiple filtering criteria such as annealing temperature, GC content, product length etc. was previously successfully used to develop SSR markers in other species [37,38]. Microsatellite loci abundance increases with genome size, but as species genome size increases, PCR amplification efficiency often decreases due to the dilution effect or multiple/non-specific priming sites. The proportion of available target DNA decreases in a template DNA volume, and the amount of non-specific primer binding increases [29]. This poses a major challenge in designing SSRs in *T. cinerariifolium* and other species with large genomes by the traditional laboratory method for screening genomic or enriched libraries. Next generation sequencing allows for rapid and cost-effective development of microsatellite markers in non-model species, although it should be noted that this method can still be costly and time-consuming in species with large genomes compared to other species [39]. The sequences of newly developed SSR markers were deposited into GenBank under accession numbers MW498263 to MW498279 (Table 2).

The analysis performed in MicroChecker revealed no evidence of scoring errors due to stuttering or large allele dropout. The presence of null alleles was detected at four loci (TcUniZg007, TcUniZg009, TcUniZg020 and TcUniZg037). A total of 94 alleles were detected ranging from 3 (TcUniZg020, TcUniZg023 and TcUniZg032) to 13 (TcUniZg006) with an average of 5.53 alleles per locus. Values of expected heterozygosity ranged from 0.197 (TcUniZg020) to 0.864 (TcUniZg008) with an average value of 0.545. Observed heterozygosity ranged from 0.083 (TcUniZg020) to 0.708 (TcUniZg008) with an average value of 0.483 (Table 3). Similar values of observed heterozygosity were previously recorded in *H. italicum*, another outcrossing perennial species characterized by extensive gene flow [32]. Only TcUniZg037 showed significant deviation from Hardy-Winberg equilibrium (*p* < 0.05). The probability of identity varied between loci from 0.043 (TcUniZg008) to 0.668 (TcUniZg020) with combined non-exclusion probability of identity for all loci of 1.94 × 10^−11^. The polymorphic information content varied from 0.178 (TcUniZg020) to 0.824 (TcUniZg008) with an average value of 0.492. Of the 17 markers tested, eight were classified as moderately polymorphic (*PIC* > 0.44), while two microsatellite markers were classified as highly polymorphic (*PIC* > 0.70) [40]. A low *PIC* was observed at the microsatellite loci TcUniZg004, TcUniZg013, TcUniZg020 and TcUniZg037 (*PIC* < 0.29), where only a small number of alleles were present (*N_a_* < 5), suggesting that these loci are not suitable for use in genetic diversity analysis of the species (Table 3).

### 2.3. Preliminary Genetic Diversity Study of Dalmatian Pyrethrum Populations

Based on the descriptive parameters, a subset of 12 developed microsatellite markers (TcUniZg005, TcUniZg006, TcUniZg008, TcUniZg010, TcUniZg012, TcUniZg013, TcUniZg014, TcUniZg017, TcUniZg019, TcUniZg023, TcUniZg032, and TcUniZg038) was used in the preliminary genetic diversity study of three Dalmatian pyrethrum natural populations (Table 4). Four microsatellite markers found to have null alleles (TcUniZg007, TcUniZg009, TcUniZg020, and TcUniZg037) and one marker with a very low PIC value (TcUniZg004) were not used in this study.

**Figure 2:**
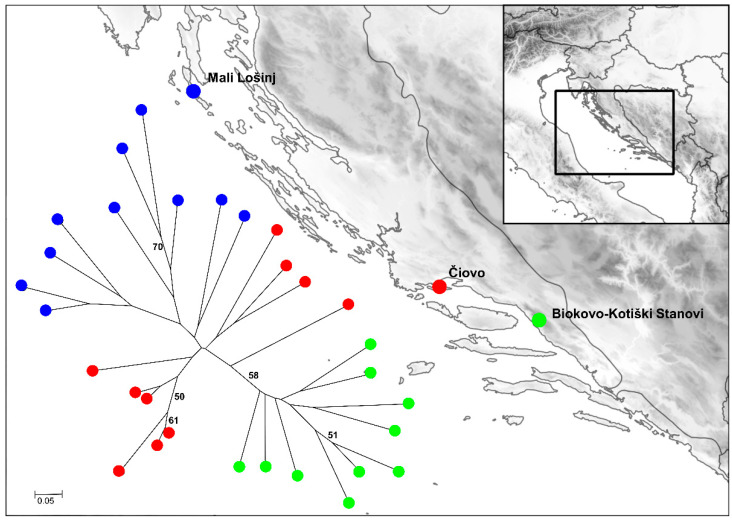
NJ tree based on *D_PSA_* between 30 Dalmatian pyrethrum individuals from three distinct populations. Bootstrap values greater than 50% are indicated on the tree.

The highest *D_PSA_* (0.875) was recorded between several pairs of individuals from Mali Lošinj and Mt. Biokovo populations, and lowest between two individuals from Čiovo population. The average *D_PSA_* between 30 individuals was 0.557. Based on the genetic distance matrix of 30 Dalmatian pyrethrum samples, a Neighbor-joining tree was constructed (Figure 2), showing grouping of sampled individuals into their populations of origin. Only the separation of Mt Biokovo clade is supported with a bootstrap value larger than 50%. Similar results were obtained in previous study employing AFLP markers, where three populations from Mt Biokovo differentiated from the rest of the Croatian populations, possibly due to geographic and genetic isolation [15]. These preliminary results on the genetic diversity of Dalmatian pyrethrum are encouraging for a future full-scale study across the entire distribution range of the species with a larger sample size.

## 3. Materials and Methods

### 3.1. Plant Material

Leaves for the NGS were sampled from Dalmatian pyrethrum plant, Accession No. MAP02806; the Collection of Medicinal and Aromatic Plants, Zagreb, Croatia as available at the Croatian Plant Genetic Resources Database—https://cpgrd.hapih.hr (accessed on 14 February 2022) grown in the Botanical Garden of the Faculty of Science, University of Zagreb. For initial testing of newly developed SSR markers, five plant samples, each originating from distinct population (MAP02769, MAP02797, MAP02799, MAP02813 and MAP02809 from the Collection of Medicinal and Aromatic Plants) were collected in 2018 from a field experiment laid out in randomized complete block design at the experimental field station of the Institute for Adriatic Crops and Karst Reclamation in Kaštel Stari, Croatia. For additional testing and subsequent characterization of SSRs, 20 samples from one population (MAP02806) were collected from the same field experiment.

To evaluate the effectiveness of the developed SSR markers in the genetic diversity study of Dalmatian pyrethrum, 10 samples of *T. cinerariifolium* were collected at each of the three geographically distinct sites in Croatia (Table 4). The collection of samples from natural habitats in Croatia, including Biokovo Nature Park, was approved by the authority of the Ministry of Environmental Protection and Energy of the Republic of Croatia (UP/I-612-07/17-48/47, 517-07-1-1-1-17-6; 21 April 2017).

### 3.2. DNA Isolation

DNA for NGS was isolated from 100 mg of fresh plant tissue using the OmniPrep™ for Plant Kit (G-Biosciences, St. Louis, MO, USA). The manufacturer’s instructions were followed except that 1% 2-mercaptoethanol and 1% polyvinylpyrrolidone (PVP) were added to the genomic lysis buffer. The concentration and purity of the isolated DNA were measured using the NanoPhotometer P300 spectrophotometer (Implen, Munich, Germany) and Qubit™ Fluorometer (Invitrogen, Carlsbad, CA, USA). The extracted DNA is stored at the Laboratory of genetic diversity, phylogeny and molecular systematics of plants of the Faculty of Science, University of Zagreb under the accession number ZAGR 47776.

Total genomic DNA of samples for both testing and characterization of the microsatellite loci, and genetic diversity study was isolated from 25 mg of silica gel-dried plant leaf tissue using the GenElute™ Plant Genomic DNA Miniprep Kit (Sigma-Aldrich^®^, Steinheim, Germany). Prior to isolation leaf tissue was ground to a fine powder using the TissueLyser II (Qiagen^®^, Hilden, Germany). The concentration and purity of the isolated DNA were measured using the NanoPhotometer P300 spectrophotometer (Implen, Munich, Germany).

### 3.3. Next Generation Sequencing, DNA Assembly and SSR Identification

The sequencing library was prepared by random fragmentation of the DNA sample, followed by 5′ and 3′ adapter ligation using the TruSeq DNA PCR Free kit (Illumina^®^, San Diego, CA, USA). Fragmentation was verified using the High Sensitivity DNA kit on the Agilent 2100 Bioanalyzer (Agilent Technologies^®^, Santa Clara, CA, USA). The library was sequenced using the Illumina HiSeq X Ten sequencer at Macrogen Europe^®^. The FastQC tool was used to check the quality of the raw sequences and the possible presence of the sequence adapters [41]. Raw sequencing reads were deposited in Sequence Read Archive (SRA) of NCBI under accession number SRX13877838.

Two approaches were used in the development of microsatellite markers for *T. cinerariifolium*. The first approach was based on *de novo* assembly of 20% of the total sequencing data randomly subsampled using the reformat.sh tool, part of the BBMap/BBTools package [42]. The assembly was performed on this set of data using the *de novo* Assembly algorithm of the CLC Genomics Server ver.20.0.2 (Qiagen Bioinformatics, Aarhus, Denmark) with default parameters and high similarity fraction (0.95) and mapping option ON.

In the second approach, evidence of contiguity from paired end reads were used. The complete dataset was searched for pairs with overlapping evidences using the bbmerge tool from the BBMap/BBTools package, which were then deduplicated using the dedupe.sh tool from the BBMap/BBTools package [42]. The obtained contigs from both approaches were re-mapped with the full set of sequencing data using the Map Reads to Contigs tool from CLC Genomics Server to obtain coverage information. Screening for possible repeat associated annotations of contigs was performed using RepeatMasker ver. 4.1.0 against combined Dfam 3.1 and RepBase (20170127) element databases [43].

Sequences obtained from both approaches were combined. To find positions of microsatellite repeats in the contigs, the MISA tool script [44] was used with search parameters for motif length of 2–6 nucleotides and with defined minimum microsatellite repeat lengths (2–8, 3–6, 4–5, 5–4, 6–4). The sequences were further analysed by BLASTn algorithm against the locally formatted database of draft genome of *T. cinerariifolium* [23] for a set of occurrences using the locally installed BLAST 2.10.1+ executable [45].

Sequences were filtered for further characterization of SSR markers and testing in natural populations of Dalmatian pyrethrum using the following selection criteria: (1) loci with compound microsatellite repeats were removed; (2) sequences with any repetitive element occurrences were removed; (3) reads mapped with multi-mapping occurrences were removed; (4) sequences with an average coverage of mapped reads close to the expected sequencing coverage depth and a length of more than 130 nucleotides were considered; (5) sequences with one or two occurrences in the draft genome of *T. cinerariifolium* were considered; (6) dinucleotide and trinucleotide motifs with longer repeats were preferred; and (7) sequences with guanine-cytosine (GC) content closer to the GC-content of the genome (43 ± 10%) were preferred. Sequences that met the listed requirements were used for primer design using Primer3 [46,47] with the parameter ‘one primer for each seq’ to obtain a single primer pair for each locus.

### 3.4. Testing and Characterization of Developed SSR Markers

The selected microsatellite primers were first tested on five samples from different populations (MAP02769, MAP02797, MAP02799, MAP02813 and MAP02809 from the Collection of Medicinal and Aromatic Plants). Polymorphic microsatellite markers with high amplification rate were further tested on 20 DNA samples from one population (MAP02806) using a tailed primer protocol [48]. The 20 μL of reaction mix contained 0.06 µM of tailed forward SSR primer, 0.25 µM of reverse SSR primer, 0.25 µM of FAM. NED, VIC or PET 5′ labeled M13 primer (5′-TGTAAAACGACGGCCAGT-3), 1 × PCR buffer with 1.5 mM MgCl2, 0.2 µM of each dNTP, 0.5 U Taq™ HS DNA polymerase (Takara^®^ Bio Inc., Shiga, Japan), and 5 ng of template DNA. A PCR protocol with an initial touchdown cycle (94 °C for 5 min; 5 cycles of 45 s at 94 °C, 30 s at 60 °C, which was lowered by 1 °C in each cycle, and 90 s at 72 °C; 25 cycles of 45 s at 94 °C, 30 s at 55 °C, and 90 s at 72 °C; and 8-min extension step at 72 °C) was used [49]. Fluorescently labeled PCR products were detected on an ABI 3730XL (Applied Biosystems^®^, Foster City, CA, USA) by the service Fragment Analysis (Macrogen Europe^®^). Allele sizes of PCR products were estimated using GeneMapper 4.0 software (Applied Biosystems^®^, Foster City, CA USA).

For each microsatellite locus, the average number of alleles per locus (*N_a_*), observed (*H_E_*) and expected (*H_O_*) heterozygosity, the probability of deviations from Hardy-Weinberg equilibrium, and the inbreeding coefficient (*F_IS_*) were calculated using GENEPOP v. 4.4 [50]. Sequential Bonferroni corrections [51] were applied when multiple statistical tests were performed in SAS. For each locus, the frequency of null alleles (*F_null_*) was calculated, and loci were analysed for scoring errors and allelic dropout using MicroChecker v. 2.2.3 [52]. Polymorphic information content (*PIC*) and the probability of identity (*PI*) were calculated using Cervus v. 3.0.7 [53].

### 3.5. Preliminary Study of Dalmatian Pyrethrum Genetic Diversity

To evaluate effectiveness of developed SSR markers in differentiation of Dalmatian pyrethrum populations, the same tailed primer protocol as described in previous section was utilized. The distance based on the proportion of shared alleles (*D_PSA_*) [54] between all pairs of individuals from three populations based on 12 microsatellite loci was calculated using MICROSAT [55]. Cluster analysis using the neighbor-joining (NJ) method with 1000 bootstraps was performed with the programs NEIGHBOR and CONSENSE (PHYLIP package) to construct a dendrogram [56].

## 4. Conclusions

With ever increasing urbanization in the Croatian coastal region Dalmatian pyrethrum populations are faced with the risk of habitat loss and subsequent decline in species richness. Newly developed SSR markers will be a powerful tool in the assessment of genetic diversity and structure of the species. In the future, these markers will serve as genetic background control in genome-wide association studies (GWAS) based on DArTseq-derived SNP markers in the hope of identifying quantitative trait nucleotides (QTNs) associated with variation in pyrethrin content and composition.

## Figures and Tables

**Table 1:** Summary of de novo assembly and SSR mining.

Category	First Approach	Second Approach	Combined Results
Total number of contigs examined	923,207	5,986,468	6,909,675
Total size of examined contigs (bp)	409,503,869	1,311,396,942	1,720,900,811
Total number of identified SSRs	11,652	23,904	35,556
SSR containing contigs	9973	21,407	31,380
Contigs containing more than one SSR	1267	2005	3272
GC content (%)	34.34	34.41	35.89

**Table 2:** List of 17 newly developed SSR markers for *T. cinerariifolium*.

Locus Name	GenBank Acc. No.	Primer Sequence (5′ to 3′)	Repeat Motif	Size (bp)
TcUniZg004	MW498263	F*: TGATCTTTTAAATTTGGAACTGAA	(GT)_10_	248–256
		R: GAAAGCTTCCTCTACCTCCTTG		
TcUniZg005	MW498264	F: CCAGATCATTTACTTAAAATGGAACA	(AC)_8_	217–223
		R: TACAACACTGGTGGCGTCAT		
TcUniZg006	MW498265	F: CGACGGTTGGTGTGTGTATC	(GA)_10_	224–280
		R: CCATACGTGTCTCTTTCTCTTTGA		
TcUniZg007	MW498266	F: GCTTCACATGGTTCGTCTCTG	(CA)_8_	191–203
		R: GCTTCACATGGTTCGTCTCTG		
TcUniZg008	MW498267	F: TGCGATGATGATGATTGAGAG	(GT)_11_	116–146
		R: ATGGCAGAACATTCAACACAA		
TcUniZg009	MW498268	F: TCTCCTTCTTCCTCCTGCAA	(CA)_11_	106–118
		R: GGATGTTTGTTGTGTTCATTGG		
TcUniZg010	MW498269	F: CATACCTCCGCCCTTGATTA	(TG)_8_	180–194
		R: CCAAGACCCACTTTTTGGTG		
TcUniZg012	MW498270	F: TCATCATCAACAAAATATCCAAGAA	(CA)_10_	244–254
		R: CCACCGACCACCTCATAATC		
TcUniZg013	MW498271	F: ACATAACGTCGGAGGCATCA	(TA)_8_	216–222
		R: TGAGTTGGGTGCGTTACAAA		
TcUniZg014	MW498272	F: AGCATAGACTGACTGTTCCTTCA	(TG)_12_	216–230
		R: CCATATTCATCACAGCCTACGA		
TcUniZg017	MW498273	F: AAGGCTGCGCTTCTTAACAG	(TA)_10_	258–274
		R: TAGCCATGCCTGGGTACTTC		
TcUniZg019	MW498274	F: AATGTGTGACTAATGGTCCTCAGA	(TA)_8_	116–124
		R: TGTTACTTAATTATAACATGCGGCCTA		
TcUniZg020	MW498275	F: ACCACCAATACAAATACACCTTC	(CA)_7_	113–117
		R: GCAGAGGCTCGAGCTAGGAC		
TcUniZg023	MW498276	F: CACAAATCCTTCACCTGTCAAA	(AC)_9_	240–250
		R: GCCAGTGGCAGAAGAGAAGT		
TcUniZg032	MW498277	F: GAAATCAAGTGCGGATACGA	(CAT)_8_	106–115
		R: TTTCCATATTGTGTTTTGGGTTC		
TcUniZg037	MW498278	F: GGACGGGATTACAGAAGGTG	(CAA)_7_	249–258
		R: TCGACCTCATTATGCTGCTG		
TcUniZg038	MW498279	F: GGAGCCAAATACTAGCCTTCAA	(TTG)_6_	151–163
		R: CGTTAGTCATCCGTGAGCAA		

* Each forward primer had M13 tail (TGTAAAACGACGGCCAGT) at the 5′ end.

**Table 3:** Characterization of developed microsatellite markers of *T. cinerariifolium* based on molecular analysis of 20 samples from Hvar population.

Locus	*N_a_*	*H_O_*	*H_E_*	*F_IS_*	Sign	*P_null_*	*PIC*	*PI*
TcUniZg004	4	0.167	0.235	0.29	ns	-	0.219	0.605
TcUniZg005	4	0.304	0.449	0.322	ns	-	0.384	0.37
TcUniZg006	13	0.542	0.633	0.145	ns	-	0.605	0.158
TcUniZg007	5	0.333	0.505	0.339	ns	0.091	0.432	0.318
TcUniZg008	11	0.708	0.864	0.18	ns	-	0.824	0.043
TcUniZg009	6	0.583	0.774	0.246	ns	0.111	0.716	0.098
TcUniZg010	6	0.667	0.757	0.12	ns	-	0.697	0.11
TcUniZg012	5	0.391	0.542	0.277	ns	-	0.485	0.266
TcUniZg013	4	0.375	0.323	−0.16	ns	-	0.288	0.495
TcUniZg014	6	0.542	0.531	−0.021	ns	-	0.488	0.263
TcUniZg017	7	0.542	0.704	0.23	ns	-	0.639	0.146
TcUniZg019	5	0.583	0.72	0.19	ns	-	0.654	0.137
TcUniZg020	3	0.083	0.197	0.576	ns	0.111	0.178	0.668
TcUniZg023	3	0.458	0.433	−0.059	ns	-	0.35	0.405
TcUniZg032	3	0.5	0.592	0.155	ns	-	0.506	0.25
TcUniZg037	4	0.125	0.299	0.582	*	0.146	0.266	0.528
TcUniZg038	5	0.542	0.702	0.228	ns	-	0.634	0.15

*N_a_*—number of alleles; *H_O_*—observed heterozygosity; *H_E_*—expected heterozygosity; *F_IS_*—inbreeding coefficient; Sign—significant deviations from Hardy-Weinberg equilibrium after sequential Bonferroni corrections: ns—non-significant; *—*p* < 0.05; *P_null_*—null allele frequency; *PIC*—polymorphic information content; *PI*—probability of identity.

**Table 4:** Sampling sites of Dalmatian pyrethrum for preliminary genetic diversity study.

Accession Number ^a^	Location	Elevation (m)	Latitude ^b^	Longitude ^b^
MAP02814	Mali Lošinj	39	44.574	14.420
MAP02807	Čiovo	211	43.498	16.303
MAP02809	Biokovo—Kotiški Stanovi	1350	43.314	17.062

^a^ Accession number from The Collection of Medicinal and Aromatic Plants, as available at the CPGRD (https://cpgrd.hapih.hr (accessed on 14 February 2022)); ^b^ Latitude and longitude are expressed in decimal degrees.

## Data Availability

The datasets generated during and/or analyzed during the current study are available from the corresponding author on reasonable request.
